# Risk factors for asymptomatic malaria infections from seasonal cross-sectional surveys along the China–Myanmar border

**DOI:** 10.1186/s12936-018-2398-y

**Published:** 2018-07-04

**Authors:** Yan Zhao, Jie Zeng, Yonghong Zhao, Qingyang Liu, Yang He, Jiaqi Zhang, Zhaoqing Yang, Qi Fan, Qinghui Wang, Liwang Cui, Yaming Cao

**Affiliations:** 1 Department of Immunology, College of Basic Medical Sciences, China Medical University, Shenyang, 110122 Liaoning China; 2 Department of Pathogen Biology and Immunology, Kunming Medical University, Kunming, China; 3 Dalian Institute of Biotechnology, Dalian, Liaoning China; 4 Department of Entomology, Pennsylvania State University, University Park, State College, PA 16802 USA

**Keywords:** Asymptomatic infection, *Plasmodium*, Risk factor, Malaria, Myanmar

## Abstract

**Background:**

Border malaria, a shared phenomenon in the Greater Mekong Sub-region of Southeast Asia, is a major obstacle for regional malaria elimination. Along the China–Myanmar border, an additional problem arose as a result of the settlement of internally displaced people (IDP) in the border region. Since asymptomatic malaria significantly impacts transmission dynamics, assessment of the prevalence, dynamics and risk factors of asymptomatic malaria infections is necessary.

**Methods:**

Cross-sectional surveys were carried out in 3 seasons (March and April, July and November) and 2 sites (villages and IDP camps) in 2015. A total of 1680 finger-prick blood samples were collected and used for parasite detection by microscopy and nested RT-PCR (nRT-PCR). Logistic regression models were used to explore the risk factors associated with asymptomatic malaria at individual and household levels.

**Results:**

The prevalence of asymptomatic *Plasmodium* infections was 23.3% by nRT-PCR, significantly higher than that detected by microscopy (1.5%). The proportions of *Plasmodium vivax, Plasmodium falciparum* and mixed-species infections were 89.6, 8.1 and 2.3%, respectively. Asymptomatic infections showed obvious seasonality with higher prevalence in the rainy season. Logistic regression analysis identified males and school children (≤ 15 years) as the high-risk populations. Vector-based interventions, including bed net and indoor residual spray, were found to have significant impacts on asymptomatic *Plasmodium* infections, with non-users of these measures carrying much higher risks of infection. In addition, individuals living in poorly constructed households or farther away from clinics were more prone to asymptomatic infections.

**Conclusions:**

Sub-microscopic *Plasmodium* infections were highly prevalent in the border human populations from IDP camps and surrounding villages. Both individual- and household-level risk factors were identified, which provides useful information for identifying the high-priority populations to implement targeted malaria control.

## Background

In the past decades, intensive malaria interventions have resulted in a dramatic decline in global malaria morbidity and mortality. However, the global burden of malaria is still enormous: in 2016 there were about 216 million cases resulting in ~ 445,000 deaths [1]. The Global Technical Strategy for Malaria 2016–2030 was endorsed by World Health Assembly, calling for a reduction in global incidence and mortality of malaria by at least 90% by 2030 [2]. This ambitious plan should include all types of malaria infections, including severe and complicated, mild and uncomplicated, and asymptomatic infections [3, 4]. Compared with asymptomatic malaria, acute malaria infections are often quoted as the “tip of an iceberg”, while the overwhelming majority of malaria infections are asymptomatic as identified by molecular methods with increasing sensitivities [5–8]. Asymptomatic carriers of malaria are prevalent in both low- and high-endemicity regions and they are important reservoirs for sustaining malaria transmission because they persist for long time and harbour gametocytes that are infectious to *Anopheles* mosquitoes [9–15]. With regard to infectivity of sub-microscopic infections to mosquitoes, some studies have even reported that parasites from asymptomatic individuals were even more infectious to vectors than those from symptomatic cases [16–18]. Since the prevalence, dynamics and transmissibility of asymptomatic malaria vary geographically and are influenced by complex factors involving parasites, hosts and environments [19, 20], identification of these factors is particularly important for malaria control.

In the Greater Mekong Sub-region (GMS) of Southeast Asia, malaria distribution is heterogeneous and often concentrated along international borders [21–24]. Border regions are not only difficult to access, but human migration across the porous borders also poses further danger of malaria re-introduction [25]. Border malaria, a shared phenomenon of the GMS, is a major challenge for achieving the regional malaria elimination goal by 2030 [26]. It requires coordinated control efforts from neighbouring countries. Recently, the asymptomatic *Plasmodium* reservoir has been reported to be considerable, even in low-transmission settings along the international borders in GMS, including Thailand–Cambodia, Thailand–Myanmar and China–Myanmar borders [8, 27, 28]. Within the GMS, Myanmar has the highest malaria burden. In addition, as a consequence of internal military conflict, refugees and internally displaced people (IDP) rushed to and settled down along the international borders. The overcrowded and poor living conditions in these camps increase the risk of infectious diseases [29–31]. In the IDP camps and the nearby villages of Kachin State at the China–Myanmar border, previous surveys have detected continued malaria transmission and observed *Plasmodium vivax* outbreaks in recent years [32]. The potential spillover of disease outbreaks to neighbouring communities demands strengthened control efforts and close surveillance.

With continued transmission of malaria in the China–Myanmar border, this study wanted to address the potential of asymptomatic *Plasmodium* infections as important reservoirs. The use of highly sensitive molecular detection methods allowed the detection of almost 20% of local residents as carriers of asymptomatic *Plasmodium* infections [8]. In the present study, further efforts were undertaken to study the seasonal dynamics and risk factors of asymptomatic malaria infections in the IDP camps and surrounding villages. Knowledge gained from this study may help identify a potential bottleneck transmission season and high-risk human populations to implement targeted radical elimination practices.

## Methods

### Ethical consideration

The study protocol was reviewed and approved by institutional review boards of China Medical University, China, Pennsylvania State University, USA and the local Health Bureau of Kachin State, Myanmar. Before carrying out the study, all adult participants or legal guardians of children voluntarily signed the informed consent.

### Study sites

**Fig. 1:**
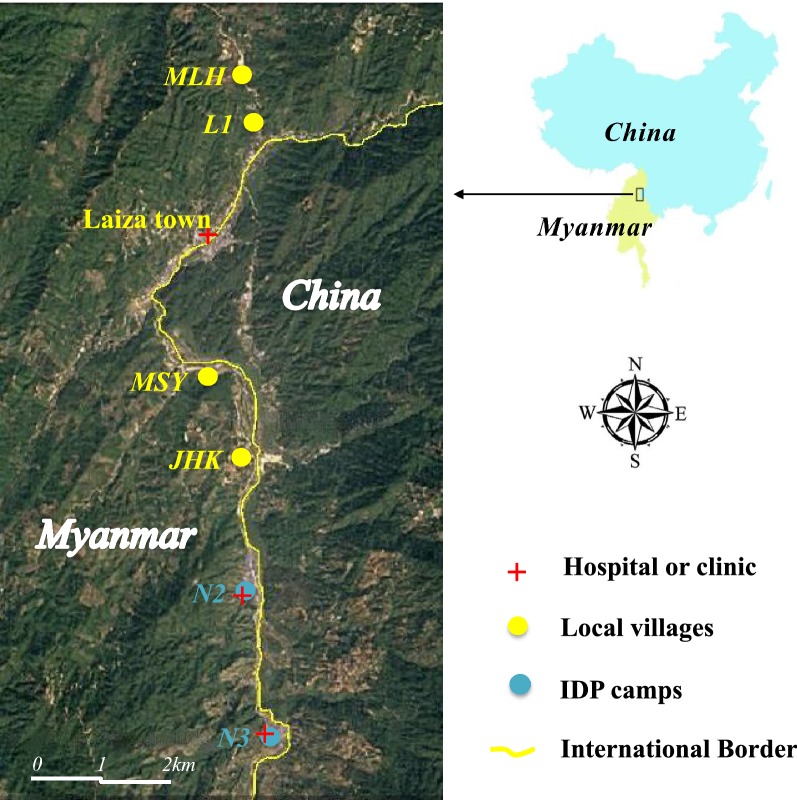
Map of study area. All villages and IDP camps in the area have been mapped as yellow and blue patches, respectively. Clinics are marked by red crosses

This study was conducted in 4 villages (MLH, L1, MSY, JHK) and 2 IDP camps (N2 and N3) near Laiza township (97.56°E and 24.75°N), Kachin State, Myanmar, along the China–Myanmar border area (Fig. 1). The study sites are located in the same valley area with an elevation ranging from 200 to 550 m above sea level. In each of the IDP camps, there is a health clinic providing routine health services to the camp residents, while the 4 villages are served by a clinic located in Laiza town. This region has a sub-tropical climate with November to April the dry and cold months, and May to October the warm and humid months. Malaria transmission in this region is perennial and dynamic, concentrated in the rainy season [33]. *Anopheles minimus* s.l. is the predominant vector species complex, and adult *Anopheles* density on average is higher in villages than IDP camps [32].

### Sample and data collection

Sample size required to detect the prevalence of asymptomatic infection in the survey sites was calculated as n = (μ^2^)(π)(1 − π)/δ^2^, using an expected asymptomatic malaria prevalence for the border (π) of 20% [8]; μ statistic of 1.96 for 95% confidence interval (CI) and marginal error (δ) of 2%. Three cross-sectional surveys were conducted in 2015 to assess malaria prevalence in different seasons: March and April, July and November to represent the pre-peak (early), peak, and post-peak (late) seasons, respectively. To encourage participation in this study, a team comprised of nurses from the local Health Bureau visited the villages and camps to inform village leaders and camp authorities about the study. During the surveys, trained local nurses and medical technologists visited the households in the villages and IDP camps to obtain informed consent from the head of each household prior to the interview and sample collection. Instructed questionnaires were used to collect individual demographic and behavioural (gender, age, occupation, malaria history, bed net usage) and household information (education of the household head, major building material of the house, its elevation and spatial location relative to landmarks such as road, forest, water body, border, and clinic, as well as the use of indoor residual spray (IRS). Age was arbitrarily grouped into 2 categories: ≤ 15 and > 15 years, whereas occupation was grouped into 4 categories: students (pre-school and school children), office and in-house workers (office staff, teachers and housewives), soldiers, and farmers. The information on characteristics of households was obtained mainly from heads of household. Housewives were respondents if the heads were away at work. The distance to the indicated locations was calculated in ArcGIS 10.2. Major building material of the house was used as a proxy for the economic status of the family, as economically better families would invest on improvement of house structure with bricks in contrast to the poor living in houses or huts made of naturally available, cheaper materials such as wood or bamboo [34]. During the visit, axillary temperature was measured and those with temperature ≥ 37.5 °C or a history of fever during the prior 24 h were referred to the nearest local health staff or clinic to receive adequate treatment and follow-up as clinically appropriate. Finger-prick blood was used to make thick and thin smears, which were air dried, transported to a nearby field laboratory, and stained with 10% Giemsa. In addition, 100 μL of finger-prick blood were collected in tubes containing EDTA and transferred on ice to the laboratory, where it was mixed with 200 μL Trizol reagent and stored at − 80 °C. The samples were shipped on dry ice to China Medical University for laboratory studies.

### Parasite detection by microscopy and nested RT-PCR

Thick and thin blood films were screened for the presence of malaria parasites using light microscopes with 100× oil immersion lens. Two microscopists examined all slides, and when discrepancy occurred, a third microscopist read the slides and the results were combined. For RNA extraction, 1 mL of Trizol reagent was added to the frozen sample containing 0.1 mL of blood. Total RNA was isolated according to the manufacturer’s protocol and dissolved in 30 μL RNase-free water. One microgram of each RNA sample was used to synthesize cDNA using the Takara RNA PCR kit (AMV) version 3.0 (Takara, Japan). Parasite detection was performed by nested RT-PCR (nRT-PCR) targeting the 18S rRNA using an established protocol [8], which showed a limit of detection of 0.01 parasites/μL of blood.

### Statistical analysis

Maps were generated using Google Earth. Statistical analysis was performed using SPSS Statistics 19.0. For risk factor analysis, both univariate and multivariate mixed effects logistic regression models were applied. First, explanatory variables were tested individually in univariate analysis. Then, all factors with *P *< 0.25 were selected to be involved in the following multivariate analysis [35, 36]. Potential risk factors were analysed at both individual and household levels, and only those with *P *< 0.05 in multivariate analysis were considered to be the risk factors. Individual-level, independent variables included gender, age, occupation, malaria history, and bed net usage. Household-level factors included education of household head, building material of the house, IRS usage and location elevation. In addition, spatial location relative to landmarks was acquired for each household and considered household-level variables for analysis. For descriptive analyses, χ^2^ test or Fisher’s exact test was used. Asymptomatic malaria prevalence for the predictive factors was compared between the rainy and dry seasons, and between villages and IDP camps using χ^2^ test, and odds ratio (OR) and adjusted odds ratio (AOR) were calculated. *P* < 0.05 was considered statistically significant.

## Results

### Characteristics of the study population

**Fig. 2:**
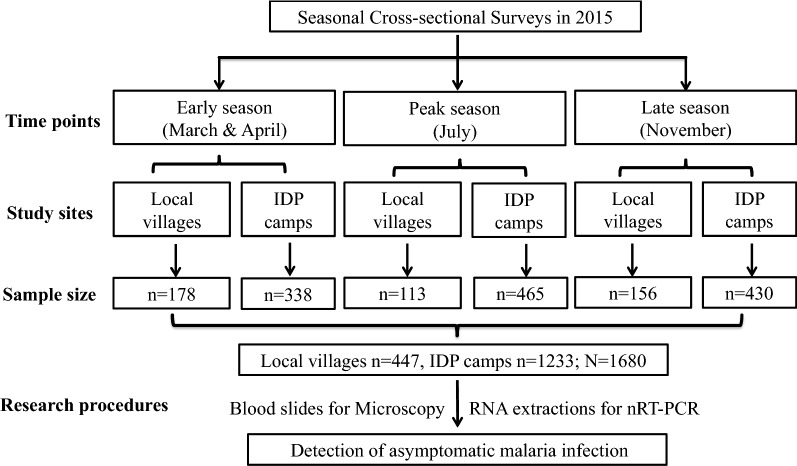
Flow chart of sample collection and research procedures. *n* number of samples, *N* total number of samples

A census prior to the surveys identified the population size in the 4 study villages as 1436. The IDP camps were established in 2011 as the result of Myanmar civil war with an initial population of ~ 7000, which was increased to 10,402 in 2015 with populations migrated from more interior areas of Kachin. Using the prevalence of ~ 20% of asymptomatic infections determined using a sensitive molecular method [8], the sample size to detect malaria parasite infection prevalence was determined to be 1537. Thus, the sample size was expanded to include 1680 healthy individuals without fever and malaria-related symptoms from 773 households, which represented 38.4% (773/2014) of the total households (Fig. 2). Three cross-sectional surveys were conducted in early (n = 516), peak (n = 578) and late (n = 586) seasons. Though the census identified 48.8% of the population as males, many adult males could not be reached as they were required to be enlisted. Therefore, the surveyed population was female-biased (70.3%). The median age of the volunteers was 33 years (95% CI 31–34), with more than 70% participants in > 15 age groups in each season. The majority of the participants were office and in-house workers (54.6%), followed by students (28.3%). When asked about their malaria histories, 3.6% of the participants self-admitted that they had malaria within the last year. With regard to preventive measures targeting vectors, participants were specifically asked for the use of bed nets. The earlier study established that bed net ownership in the study community was close to 100%, but usage was ~ 75%. The current surveys identified that less than half (45.1%) of the participants used bed nets in previous nights (Table 1). Of the 773 households surveyed, 35.4% had IRS during the previous month. The majority of the heads of the households had primary (34.9%) or middle school (63.0%) levels of education. With regard to the house structures and building materials, the majority of the houses were built using wood and bamboo as the main construction materials (95.2%), whereas only 5.2% of the houses were constructed using bricks (Table 2).

**Table 1:** Demographics of the study population [n (%)]

Characteristics	Early season	Peak season	Late season	Total
Number of participants (% male)	516 (26.0)	578 (36.2)	586 (26.6)	1680 (29.7)
Age groups (years)				
≤ 15	76 (14.7)	165 (28.6)	165 (28.2)	406 (24.2)
> 15	440 (85.3)	413 (71.4)	421 (71.8)	1274 (75.8)
Occupation				
Students	96 (18.6)	187 (32.4)	192 (32.8)	475 (28.3)
Office and in-house workers	309 (59.9)	285 (49.3)	323 (55.1)	917 (54.6)
Soldiers	19 (3.7)	25 (4.3)	17 (2.9)	61 (3.6)
Farmers	92 (17.8)	81 (14.0)	54 (9.2)	227 (13.5)
Malaria history (in the last year)	39 (7.6)	8 (1.4)	13 (2.2)	60 (3.6)
Use of bed net	266 (51.6)	260 (45.0)	237 (40.4)	763 (45.1)

**Table 2:** Characteristics of the study households

Education of household head [N (%)]	
Illiterate	11 (1.4)
Primary school	270 (34.9)
Middle school	487 (63.0)
High school and above	5 (0.6)
Major material of house [N (%)]	
Wood/bamboo	733 (95.2)
Brick	40 (5.2)
Use of IRS [N (%)]	285 (36.9)
Elevation (meters above sea level) [median (IQR)]	312 (298–390)
Distance to the nearest forest (m) [median (IQR)]	200 (31–300)
Distance to the nearest clinic (m) [median (IQR)]	180 (80–250)
Distance to the nearest water body (m) [median (IQR)]	50 (16–190)
Distance to the nearest road (m) [median (IQR)]	15 (10–25)
Distance to the border (m) [median (IQR)]	100 (45–200)

*IQR* inter quartile range

### Asymptomatic malaria parasite prevalence

Microscopic examination of the 1680 blood smears only detected *P. vivax* infections in 25 samples, giving an overall 1.5% parasite prevalence. The prevalence of *P. vivax* infections in the peak season (2.3%) was slightly higher than that of the early season (1.7%) and late season (1.5%), but the differences were not statistically significant (Table 3). In comparison, nRT-PCR targeting the 18S rRNA gene detected 23.4% (393/1680) prevalence of asymptomatic infections. Specifically, 23 of the 25 microscopy-positive samples were confirmed by nRT-PCR, demonstrating that the majority (94.1%) of the asymptomatic infections were sub-microscopic. The 393 nRT-PCR positive samples included 352 (89.6%) *Plasmodium vivax*, 32 (8.1%) *Plasmodium falciparum*, and 9 (2.3%) mixed *P. vivax/P. falciparum* infections. *Plasmodium ovale, Plasmodium malariae* and *Plasmodium knowlesi* infections were not detected. It is noteworthy that *P. falciparum* infections were only identified in the 2 later surveys.

**Table 3:** Prevalence of asymptomatic malaria infections in different seasons detected by microscopy and nRT-PCR

Diagnosis	Parasite	Early [n (%)]	Peak [n (%)]	Late [n (%)]	Total [n (%)]	P value^1^
Microscopy	*P. vivax*	9 (1.7)	13 (2.3)	3 (0.5)	25 (1.5)	0.066
	*P. falciparum*	0	0	0	0	–
	Mixed infection	0	0	0	0	–
	Total	9 (1.7)	13 (2.3)	3 (0.5)	25 (1.5)	0.066
nRT-PCR	*P. vivax*	95 (18.4)	152 (26.3)	105 (17.9)	352 (21.0)	0.001
	*P. falciparum*	0	14 (2.4)	18 (3.1)	32 (1.9)	< 0.001
	Mixed infection	0	8 (1.4)	1 (0.2)	9 (0.5)	0.002
	Total	95 (18.4)	174 (30.1)	124 (21.2)	393 (23.4)	0.001

^1^Chi square test or Fisher’s exact test for comparison among different seasons. Significant at *P *< 0.05. Mixed infection—*P. falciparum* and *P. vivax*

**Fig. 3:**
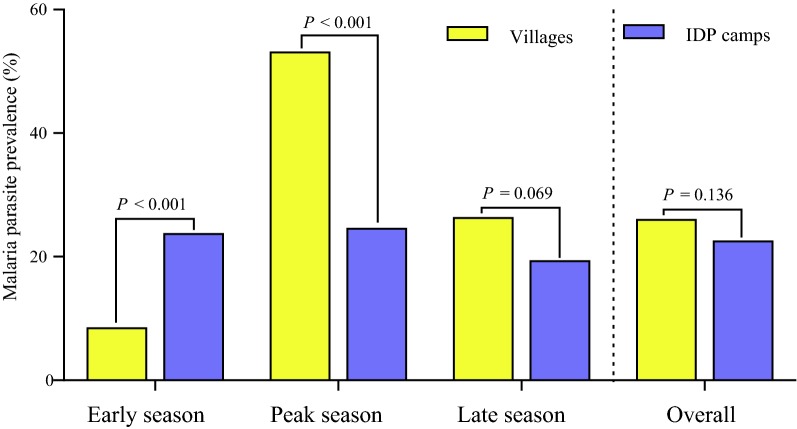
Prevalence of asymptomatic malaria carriage in different study sites identified by nRT-PCR. Chi square (χ^2^) test was used for assessing differences in asymptomatic malaria infection between village and IDP camp populations in three seasons. The infections included all species detected by nRT-PCR

In contrast to the results from microscopic examination, there were significantly seasonal differences in the prevalence of infections detected by nRT-PCR (*P *= 0.001): 18.4% of infection occurred in early season (March–April), 30.1% in the rainy season (July), and 21.2% in the late season (November) (Table 3). This seasonal variation was notably dramatic among the villagers. Prior to the rainy season, the prevalence of asymptomatic infection in the villages was relatively low at 8.43%, which sharply rose to > 50% during the peak transmission season (Fig. 3). In comparison, the prevalence of sub-microscopic infections did not show substantial seasonal variation in the IDP camps (Fig. 3). Furthermore, the comparison of the asymptomatic infections between villages and IDP camps during the different seasons revealed that before the rainy season, the prevalence of asymptomatic infection in the villages (8.43%) was significantly lower than that in the IDP camps (8.43 vs 23.7%, *P *< 0.001), whereas this trend was obviously reversed in the peak transmission season (54.1 vs 24.5%, *P* < 0.001). This trend was maintained after the rainy season, and the prevalence of asymptomatic infections in the villages (26.3%) was much higher than that in the IDP camps (19.3%) in November, though the difference was marginal (*P *= 0.069).

### Risk factors associated with asymptomatic malaria infections

Univariate and multivariate analyses were performed to identify the risk factors associated with asymptomatic malaria infections. In the univariate analysis, all potential factors were tested and only those with *P* values less than 0.25 (including gender, age, bed net use, major building material of house, IRS use, elevation, location to clinic and road) were selected and set into the final multivariate model. First, the results showed that males were significantly more likely than females to have asymptomatic infections (AOR: 1.5; 95% CI 1.16–1.97, *P *= 0.002). Second, the age group of ≤ 15 had significantly higher odds of malaria infection (AOR = 1.49, 95% CI = 1.13–1.96, *P *= 0.004). Third, the effects of vector control-based preventive measures were very remarkable. Those who did not sleep under the bed nets had more than 5 times higher odds of asymptomatic infections than those who did (AOR: 5.49, 95% CI 4.12–7.32, *P* < 0.001). People from the households that did not have IRS had 3.4 times higher odds of asymptomatic infection than those with IRS (95% CI 2.50–4.65, *P* < 0.001). Fourth, major building materials of the houses, which reflect the economic status of the family, also showed significant effect on asymptomatic infections. Residents from the houses constructed from wood or bamboo had approximately twice the odds of having *Plasmodium* infections (AOR: 1.90, 95% CI 1.15–3.14, *P *= 0.012). Last, longer distance of household to the nearest clinic (> 180 m) was also significantly associated with a higher risk of malaria infection (AOR: 2.05, 95% CI 1.53–2.75, *P* < 0.001) (Table 4).

**Table 4:** Assessment of risk factors for asymptomatic malaria infection

Predictive factors	Category	PCR positive [n (%)]	Univariate	P value^1^	Multivariate	P value^2^
			OR (95% CI)		AOR (95% CI)	
Individual level						
Gender	Male	153 (30.7)	1.73 (1.37, 2.20)	< 0.001	1.51 (1.16, 1.97)	0.002
	Female	240 (20.3)	1		1	
Age groups (years)	≤ 15	135 (33.3)	1.96 (1.53, 2.51)	< 0.001	1.49 (1.13, 1.96)	0.004
	> 15	258 (20.3)	1		1	
Bed net use	No	326 (35.6)	5.73 (4.31, 7.62)	< 0.001	5.49 (4.12, 7.32)	< 0.001
	Yes	67 (87.8)	1		1	
Household level						
Major material of house	Wood/bamboo	371 (37.0)	1.55 (0.96, 2.48)	0.072	1.90 (1.15, 3.14)	0.012
	Brick	22 (30.1)	1		1	
Use of IRS	No	337 (48.5)	3.44 (2.54, 4.66)	< 0.001	3.40 (2.50, 4.65)	< 0.001
	Yes	56 (14.7)	1		1	
Elevation of house (m)	≤ 312	250 (45.0)	1.82 (1.45, 2.30)	< 0.001	1.11 (0.83, 1.49)	0.490
	> 312	143 (27.5)	1		1	
Distance to the nearest clinic (m)	> 180	257 (54.9)	2.49 (1.97, 3.15)	< 0.001	2.05 (1.53, 2.75)	< 0.001
	≤ 180	136 (22.4)	1		1	
Distance to the nearest road (m)	≤ 15	277 (48.2)	1.55 (1.22, 1.98)	< 0.001	1.20 (0.92, 1.57)	0.189
	> 15	116 (23.2)	1		1	

*PCR* polymerase chain reaction, *OR* odds ratio, *AOR* adjusted odds ratio, *CI* confidence interval, *IRS* indoor residual spraying

^1^P value was determined in a univariate logistic regression model

^2^P value was assessed in the multivariate mixed effects logistic regression model—variables with P values < 0.25 in the unadjusted analysis were considered

### Risk factors in different seasons

To further explore whether the risk factors for asymptomatic malaria infections experience seasonal variations, the 2 surveys conducted in the dry season (March–April and November) were combined and compared with that conducted in the rainy season (July). With regard to preventive measures, those who did not use bed nets had 10.53 (95% CI 6.38–17.40; *P* < 0.001) and 3.81 times (95% CI 2.67–5.43, *P* < 0.001) higher odds of having asymptomatic infections than those who used bed nets in the rainy and dry season, respectively (Table 5). Moreover, those who did use bed nets in the rainy season had 2.28 times higher odds of having asymptomatic infections than those in the dry season (95% CI 1.72–3.03, *P* < 0.001). Similarly, those without the use of IRS had 7.78 (95% CI 4.57–13.25, *P* < 0.001) and 2.29 (95% CI 1.54–3.40, *P* < 0.001) times higher odds of infection in the rainy and dry season, respectively (Table 5), giving a rainy season/dry season risk ratio of 2.43 (95% CI 1.86–3.17, *P* < 0.001). Furthermore, larger distance (> 180 m) of the household to the nearest clinic was significantly associated with a higher risk of malaria infections in both seasons. For other factors, there were also seasonal variations. Whereas the male gender had increased odds of asymptomatic infections in the rainy season, the factor did not seem to contribute to higher infection prevalence in the dry season (Table 5). Also, people who were ≤ 15 years and lived in houses constructed with wood/bamboo, which were located in lower elevation (≤ 312 m above sea level) and closer to road (≤ 15 m) had higher odds of having asymptomatic infections only in the dry season.

**Table 5:** Assessment of risk factors for asymptomatic malaria infections in the rainy and dry seasons

Predictive factors	Category	Rainy season		Dry season		Risk ratio (95% CI)^2^
		PCR+ (%)	AOR (95% CI)^1^	PCR+ (%)	AOR (95% CI)^1^	Rainy/dry seasons
Overall		30.1		19.9		1.74 (1.38−2.19)***
Individual-level						
Gender	Male	36.8	1.61 (1.05−2.46)*	26.2	1.36 (0.96−1.92)	1.64 (1.12−2.41)*
	Female	26.3	1	17.6	1	1.67 (1.24−2.24)**
Age groups (years)	≤ 15	35.8	0.97 (0.62−1.52)	31.5	1.88 (1.32−2.67)***	1.21 (0.80−1.84)
	> 15	27.8	1	16.6	1	1.94 (1.46−2.56)***
Bed net use	No	48.1	10.53 (6.38−17.40)***	28.9	3.81 (2.67−5.43)***	2.28 (1.72−3.03)***
	Yes	8.1	1	9.2	1	0.87 (0.51−1.50)
Household-level						
Major material of house	Wood/bamboo	30.1	–	20.6	2.15 (1.16−4.0)*	1.66 (1.31−2.10)***
	Brick	31.0	1	12.9	1	3.05 (1.15−8.11)*
Use of IRS	No	42.3	7.78 (4.57−13.25)***	23.2	2.29 (1.54−3.40)***	2.43 (1.86−3.17)***
	Yes	9.7	1	11.4	1	0.84 (0.47−1.49)
Elevation of house (m)	≤ 312	35.8	1.15 (0.75−1.76)	24.7	1.56 (1.09−2.23)*	1.71 (1.26−2.31)**
	> 312	24.1	1	15.1	1	1.88 (1.30−2.71)**
Distance to the nearest clinic (m)	≤ 180	21.8	1	12.8	1	1.91 (1.31−2.77)**
	> 180	37.9	1.90 (1.16−3.11)*	28.0	2.62 (1.78−3.87)***	1.57 (1.16−2.13)**
Distance to the nearest road (m)	≤ 15	33.2	1.16 (0.76−1.78)	22.6	1.48 (1.05−2.08)*	1.21 (1.29−2.26)***
	> 15	25.0	1	15.1	1	1.87 (1.24−2.81)**

*PCR+* PCR positive, *AOR* adjusted odds ratio, *CI* confidence interval, *IRS* indoor residual spray

^1^Multivariate logistic regression model was used for mixed effected risk factors analysis

^2^Prevalence of asymptomatic malaria for predictive factors were compared between rainy season and dry seasons by using the Chi square (χ^2^) test

* *P *<0.05; ** *P *<0.01; *** *P *<0.001

### Risk factors in villages *versus* IDP camps

Further comparison of the risk factors for asymptomatic malaria infections revealed additional differences between the villages and IDP camps (Table 6). Although people who did not use bed nets from villages and camps all had increased odds of having asymptomatic *Plasmodium* infections compared to bed net users, those non-users from the camps had much higher odds of asymptomatic infections (AOR: 7.37, 95% CI 5.15–10.53, *P* < 0.001) than the non-users from the villages (AOR: 2.65, 95% CI 1.61–4.35, *P *< 0.001). Similarly, IRS use also significantly reduced the odds of asymptomatic infections in households from both villages and camps, but the effect appeared more profound in the villages (Table 6). Also, larger distance (> 180 m) of the houses to the nearest clinics was significantly associated with higher risks of malaria infections both in villages and camps. There were also village- or camp-specific risk factors. For example, lower house elevation (≤ 312 m above sea level) was significantly associated with higher odds of asymptomatic malaria infection in villages, whereas there were obvious associations of the male gender and the ≤ 15 age group with higher risks of asymptomatic infections in IDP camps.

**Table 6:** Assessment of risk factors for asymptomatic malaria infections between villages and IDP camps

Predictive factors	Category	Villages		IDP camps		Risk ratio (95% CI)^2^
		PCR+ (%)	AOR (95% CI)^1^	PCR+ (%)	AOR (95% CI)^1^	Villages/IDP camps
Overall		26.0		22.5		1.21 (0.94−1.55)
Individual-level						
Gender	Male	31.9	1.36 (0.77–2.40)	30.0	1.55 (1.06–2.26)*	1.10 (0.73−1.63)
	Female	22.4	1	19.7	1	1.18 (0.85−1.63)
Age groups (years)	≤ 15	32.5	1.47 (0.80−2.70)	33.4	1.48 (1.03−2.14)*	0.96 (0.57−1.62)
	> 15	24.5	1	18.5	1	1.43 (1.07−1.91)*
Bed net use	No	32.4	2.65 (1.61−4.35)***	37.0	7.37 (5.15−10.53)***	0.82 (0.61−1.10)
	Yes	15.1	1	7.0	1	2.34 (1.38−3.97)**
Household-level						
Major material of house	Wood/bamboo	29.56	–	22.46	–	1.46 (1.10−1.92)**
	Brick	16.92	1	–	1	–
Use of IRS	No	34.4	8.70 (3.82−19.79)***	27.2	2.67 (1.90−3.77)***	1.40 (1.06−1.85)*
	Yes	5.4	1	12.4	1	0.40 (0.18−0.91)*
Elevation of house (m)	≤ 312	33.4	3.42 (1.51−7.72)**	25.6	0.70 (0.48−1.01)	1.46 (1.08−1.97)*
	> 312	8.3	1	19.8	1	0.37 (0.19−0.70)**
Distance to the nearest clinic (m)	≤ 180	13.2	1	16.4	1	0.77 (0.49−1.24)
	> 180	35.4	2.63 (1.47−4.71)**	19.9	2.51 (1.69−3.74)***	1.29 (0.94−1.76)
Distance to the nearest road (m)	≤ 15	28.4	–	25.4	–	1.16 (0.86−1.58)
	> 15	21.8	1	17.5	1	1.32 (0.85−2.05)

*PCR+* PCR positive, *AOR* adjusted odds ratio, *CI* confidence interval, *IRS* indoor residual spray

^1^Multivariate logistic regression model was used for mixed effected risk factors analysis

^2^Infection rate of asymptomatic malaria for predictive factors were compared between local villages and IDP camps by using the Chi square (χ^2^) test

* *P *<0.05; ** *P *<0.01; *** *P *<0.001

## Discussion

Asymptomatic malaria was once thought the ‘forgotten’ malaria, but it is a major gametocyte reservoir to conduce mosquito infection and thus a major barrier for malaria elimination [37]. In addition to the complicated features of border malaria observed elsewhere, settlements for refugees and IDP along the borders add another layer of complexity to the existing border malaria problem. This study provided further evidence about the significance of asymptomatic malaria as the reservoir for continuous malaria transmission, and also identified the individual- and household-level factors associated with asymptomatic malaria infection along the China–Myanmar border. This study found increased risks of asymptomatic infections in (1) males, (2) children (≤ 15 years), (3) those who failed to use bed net and IRS, (4) those who lived in poor houses, and (5) those lived farther away from a clinic. These findings provide the needed knowledge for guiding the malaria control and elimination program in border regions and in IDP camps.

Routine microscopy with a detection limit of 50–100 parasites/μL severely underestimates the burden of asymptomatic infections [38]. In this study, nRT-PCR was confirmed to be a more sensitive technique for identification of asymptomatic *Plasmodium* infections, revealing more than 15-fold higher prevalence of asymptomatic infections than microscopy. The results were consistent with those from other parts of GMS such as Thailand and Cambodia [39–41], which showed that the majority of the asymptomatic infections were sub-microscopic. Despite that the prevalence was lower than in hyper-endemic regions in Africa [42, 43], the > 20% prevalence of vivax infection is astounding and may explain the persistence and outbreaks of acute vivax malaria in this region. Like the acute malaria incidence detected from passive case surveillance [24, 32], asymptomatic malaria along the border also exhibited seasonal variation. Compared with the dry season, the higher odds of infection during the rainy season might be related to increased density and species richness of the vectors [44, 45]. Interestingly, seasonal variation was more obvious in the villages, which might be related to the different vector ecology and control efforts in villages and camps [32].

Among the demographic factors, male gender and young children (≤ 15 years) were significantly associated with asymptomatic infections. Such a gender bias of malaria infection has been reported in other studies [19, 46–49]. In this case, males were mostly farmers and soldiers, who are engaged in more agricultural and forest-related activities, which might increase the chances of infection [50]. Age is considered one of the most important factors associated with protective immune response in malaria endemic areas. Many previous studies revealed adults are asymptomatic parasite carriers because they have acquired strong immunity from repeated exposures to malaria parasites, whereas infections in young children are symptomatic because their anti-malarial immunity is still developing [51–53]. Thus, the finding that school children (≤ 15 years) had higher odds of asymptomatic malaria infections seems contradictory to findings from Africa. Yet, this finding corroborated our earlier studies, which showed that school children had increased odds of having acute vivax malaria [32, 33]. Consistent results were also obtained from a series of recent studies revealing that school-age children were an under-recognized reservoir of malaria infection showing either higher levels of asymptomatic infection or gametocyte carriage [54–56]. Such higher prevalence of asymptomatic infections in school-age children might be resulted from increased *Plasmodium* exposure due to neglected bed net use and drug treatment. A study conducted in Papua New Guinea showed that immunity to *P. vivax* may be acquired at a younger age than that seen with *P. falciparum* [57], which might be another reason for what was observed in this study. This highlights that malaria control initiatives should specifically target school children for malaria elimination along the border.

Malaria transmission is mainly influenced by two key factors: gametocyte infectivity and vector density. Thus, effective vector-based malaria control measures such as the use of IRS and bed nets, especially long-lasting insecticide-treated nets (LLINs) are particularly important in reducing malaria incidence [58]. This study found that non-users of bed net or IRS had much higher risks of asymptomatic malaria infection in whichever seasons or sites. Moreover, the risk was much higher in rainy season than in dry season. Additionally, odds in the surrounding villages were 2.34 times higher than those in the IDP camps, which may be largely due to higher vector densities in villages [32, 44]. This result was in line with the higher asymptomatic malaria infection rates in local villages than in IDP camps in the peak transmission season [31, 32, 59]. Vector control interventions were insufficient in local villages as compared to those in the IDP camps, as the latter were helped by multiple non-governmental agencies. Bed nets in local villages were mostly conventional (> 99%), whereas > 60% of the nets in the IDP camps were LLINs, and more frequent IRS were performed in the IDP camps during the rainy season [32]. Interestingly, such a trend of asymptomatic infections was reversed in the early dry season, when mosquito density is low. Although reasons for this are not clear, it is possible that the higher asymptomatic infection rate in the IDP camps might be due to migration of human populations from more interior areas with higher malaria endemicity as the camp population grew [29, 30]. This study emphasized the importance of vector control strategies in reducing asymptomatic infections.

This study also found that household and the surrounding environmental factors affected the prevalence of asymptomatic malaria. Firstly, houses constructed with wood or bamboo increased the risks of infection, corroborating findings from other studies [60–62]. Since the house structures reflect the socio-economic status of the family, it may correlate with the use of personal protection measures [63, 64]. Interestingly, longer distance to the clinic is another factor associated with increased asymptomatic infections. The distance from residents in JHK and MSY villages to the nearest clinic is almost 1500 m, and half of the recruited populations from these 2 villages were infected. One reason might be due to potential delays for patients from these 2 villages to seek treatment, especially during the rainy season when short travel becomes prohibitive due to poor road conditions [65]. Other factors associated with the locations of the houses likely reflect the surrounding environment. For example, the higher prevalence of infections in these 2 villages might be due to their closer proximity to forest edges, where mosquito vectors associated with forested areas are more abundant as shown from the earlier study [32].

The findings of these 3 seasonal cross-sectional surveys have provided insights about persistent malaria transmission along the China–Myanmar border, which is needed for malaria elimination on both sides of the border. A sensitive molecular method verified the presence of a large submicroscopic reservoir of malaria parasites in both village and IDP populations. This study also identified risk factors associated with increased prevalence of asymptomatic infections in both populations. On the individual level, males, school children, and those who did not use bed net were the as high-risk populations. On the household level, worse living conditions, failure to use the mosquito control-based interventions and farther away from the clinic were also associated with increased risks of parasite infections. While increasing the coverage of effective malaria interventions will be the key to control border malaria, additional measures such as mass drug administration (MDA) may be considered in order to reduce the infected population. MDA with chloroquine and primaquine has been found to be successful in eliminating vivax malaria in central China [66]. Furthermore, better management of malaria cases such as the recently described ‘1-3-7’ strategy is needed in the final phase of malaria elimination [67].

This study has several limitations. First, the data collection was confined to the available residents in the households, which led to the female-biased study population and over-representation (54.6%) of office/in-house workers, which undermines the generalization of the findings. Given that males, farmers and soldiers might be the high-risk populations, future studies should ensure a better representation of the study population. Second, the use of convenience samples inevitably adds additional biases, and future study designs need to be more systematic. Third, the study did not take into account the effect of human migration on malaria transmission. It is important to find out whether and how much migration of people from more malaria-endemic interior areas to the camps contributes to the malaria transmission. Furthermore, the significance of cross-border migration for malaria importation to the neighbouring Yunnan province of China also deserves special attention. Finally, the comparison between the local villages and camps needs to be more quantitative since their accesses to malaria treatment and prevention services were different. Because the whole region is targeted for malaria elimination, provision of quality malaria control services to the entire populations will ensure achieving the goal.

## Conclusions

This study underscores the need for strong initiatives to control border asymptomatic malaria. Results from this study provide the needed knowledge to the malaria managers for defining the priority populations and areas for more intensified control efforts. Particularly, vector-based control measures such as bed nets and IRS appeared to provide significant protection against asymptomatic infections, and thus attention is needed to increase the coverage of these measures.

## Abbreviations

IDP: internally displaced people
IRS: indoor residual spraying
PCR: polymerase chain reaction
nRT-PCR: nested reverse transcription PCR
OR: odds ratio
AOR: adjusted odds ratio
95% CI: 95% confidence interval
IQR: inter quartile range
